# Brain regions vulnerable and resistant to aging without Alzheimer’s disease

**DOI:** 10.1371/journal.pone.0234255

**Published:** 2020-07-29

**Authors:** Xinyang Feng, Jia Guo, Hannah C. Sigmon, Richard P. Sloan, Adam M. Brickman, Frank A. Provenzano, Scott A. Small, for the Alzheimer’s Disease Neuroimaging Initiative

**Affiliations:** 1 Department of Biomedical Engineering, Columbia University, New York, NY, United States of America; 2 Department of Psychiatry, Columbia University, New York, NY, United States of America; 3 Department of Neurology, Columbia University, New York, NY, United States of America; 4 New York State Psychiatric Institute, New York, NY, United States of America; 5 Taub Institute for Research on Alzheimer's Disease and the Aging Brain, Columbia University, New York, NY, United States of America; Nathan S Kline Institute, UNITED STATES

## Abstract

‘Normal aging’ in the brain refers to age-related changes that occur independent of disease, in particular Alzheimer’s disease. A major barrier to mapping normal brain aging has been the difficulty in excluding the earliest preclinical stages of Alzheimer’s disease. Here, before addressing this issue we first imaged a mouse model and learn that the best MRI measure of dendritic spine loss, a known pathophysiological driver of normal aging, is one that relies on the combined use of functional and structural MRI. In the primary study, we then deployed the combined functional-structural MRI measure to investigate over 100 cognitively-normal people from 20–72 years of age. Next, to cover the tail end of aging, in secondary analyses we investigated structural MRI acquired from cognitively-normal people, 60–84 years of age, who were Alzheimer’s-free via biomarkers. Collectively, the results from the primary functional-structural study, and the secondary structural studies revealed that the dentate gyrus is a hippocampal region differentially affected by aging, and that the entorhinal cortex is a region most resistant to aging. Across the cortex, the primary functional-structural study revealed and that the inferior frontal gyrus is differentially affected by aging, however, the secondary structural studies implicated other frontal cortex regions. Together, the results clarify how normal aging may affect the brain and has possible mechanistic and therapeutic implications.

## Introduction

‘Normal aging’ refers to age-related changes in the brain that occur independent of brain disorders. For studies that set out to investigate normal human aging, many of these disorders are easily excluded. The greatest challenge has been excluding Alzheimer’s disease (AD), which is known to have a preclinical stage that occurs years before its clinical onset, and which remains undetectable by conventional clinical measures [1–3]. The lurking confound of occult AD is particularly relevant when studies appropriately attempt to include older adults at the tail end of the age-span, since the prevalence of preclinical AD is greatest during the 8^th^-9^th^ decades of life [4], reaching as high as 50%.

A technical challenge for studies interested in mapping normal aging is how best to detect its underlying pathophysiology, characterized primarily by loss of dendritic spines without neuronal death [5]. Two neuroimaging approaches should, at least in principle, be sensitive to this pathophysiological feature. As dendritic spines are known to have high metabolic needs [6], the first is ‘functional imaging’—PET mapping of fluorodeoxyglucose (FDG) or cerebral metabolic rate of oxygen consumption (CMRO2), or fMRI mapping of cerebral blood volume (CBV), cerebral blood flow (CBF), or blood-oxygen level dependent (BOLD) signal [7]—and all have been used to map functional changes in the aging brain [8–12]. For mapping age-related changes in select regions of the hippocampus, a structure implicated in normal aging [7, 13], functional imaging techniques with higher resolution are required, as previously shown using contrast-enhanced CBV fMRI [7, 14–17] or optimized BOLD fMRI [18–20]

Besides functional imaging, structural imaging is the second approach that should be sensitive to spine loss, and can generate images with submillimeter resolution from which precise measures of volume and thickness can be derived. This expectation has been validated in recent studies, showing that variables derivable from structural MRI are sensitive to dendritic spine loss [21, 22].

While both functional and structural imaging might be sensitive to spine loss, they are not necessarily specific, a concern particularly relevant to the latter since many brain elements can contribute to volume independent of spine density. In considering how to address the issue of specificity, we hypothesized that, in the absence of neuronal death, a concordant change in measures of both function and structure would represent a more specific indirect measure dendritic spine loss than each individually.

We first set out to test this hypothesis in a model system. Unilateral whisker cutting in young mice causes dendritic spine loss in select barrel cortex layers [23]. We deployed a series of new tools designed to map the functional architecture of the mouse cortex. We show that when these new processing tools are applied to CBV-fMRI, the functional organization of the barrel cortex and its different layers can, for the first time, be mapped non-invasively *in vivo*. More importantly, we validated the hypothesis that concordant changes in MRI measures of function and structure are most sentisitve to dendritic spine loss, and learned from the mouse studies how best to analyze and interpret MRI-based correlates of spine loss.

Relying on the insight from the mouse, we turned to an aging study we recently completed at Columbia University, in which we used MRI to map CBV and volume in over 100 carefully-screened cognitively normal individuals spanning 20–72 years of age. In a secondary series of studies, we set out to broaden the age-span to older subjects. To investigate an older cohort in whom we can exclude the confound of AD, we turned to the ‘Alzheimer’s Disease Neuroimaging Initiative’ (ADNI). While ADNI does not acquire high-resolution functional imaging data, it does acquire high resolution structural MRI. Guided by the results from our Columbia cohort, we derived volumetric measurements in the ADNI cohort, in participants who did not meet the current clinical and CSF biomarker criteria for AD, using a combination of CSF tau and Aβ_1–42_, with the most reliable CSF indicator of disease [24–26]. Finally, ADNI afforded us tthe opportunity to complete an exploratory longitudinal study, relying on images acquired repeatedly for over 4 years.

Collectively, the results from the primary functional-structural study, and the secondary structural studies revealed that the dentate gyrus is a hippocampal region differentially affected by aging, Across the cortex, the primary functional-structural study revealed and that the inferior frontal gyrus is differentially affected by aging, however, the secondary structural studies implicated other frontal cortex regions. Interestingly, these vulnerable regions appear to have different aging trajectories. Remarkably, both the functional-structural study and the secondary structural studies suggest the entorhinal cortex, a region most vulnerable to AD, turned out to be the region most resistant to normal aging.

## Results

### Concordant changes in functional and structural MRI reflect dendritic spine loss

**Fig 1:**
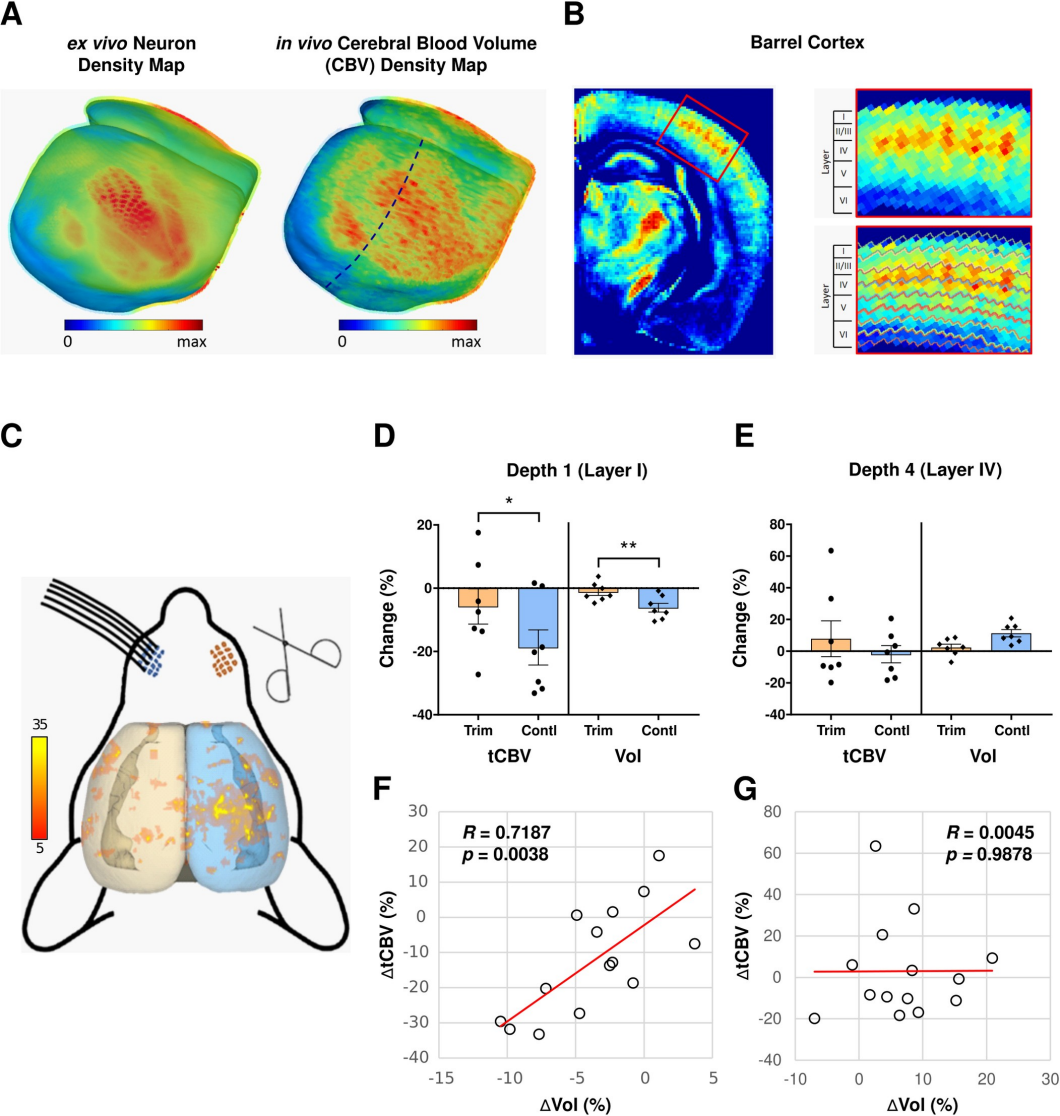
Mapping dendritic spine loss with CBV-fMRI and volumetric MRI. **(A)** 3D mouse cortical surface maps of the *ex vivo* neuron density (left image) and *in vivo* cerebral blood volume (CBV) density (right image). Cortical surface maps were generated from the template images using our proposed MouseStream software suite by projecting the mean density along each streamline (see Method for details). **(B)** CBV density map in a single coronal slice of the right hemisphere (dashed line in panel **A** right image) going through the barrel cortex (left image) and the zoom-in view of the barrel cortex (right image). The CBV density map was generated as the population average of 14 co-registered CBV scans. The whole cortex was delineated evenly into 8 depths by solving the Laplace’s Equation. Cortical layers were defined using the Allen Brain Atlas anatomical CCF v3 template. Individual barrel modules with high basal metabolism were observed at layer-IV of the barrel cortex. **(C)** Result of voxel-based analysis of percent CBV density (%CBV) across the whole cortex. The t-value distribution of CBV decrease during 30-day trimming period across cortical regions shows that the barrel cortex on the control side (right hemisphere) has significant CBV decreases (*p*<0.05, cluster>20) and the barrel cortex contralateral to the trimmed side (left hemisphere) has no clear CBV decreases. **(D)** Total CBV (tCBV) and volume changes over 30-day trimming period in the depth 1 (belongs to layer I) of the barrel cortex. At the depth that corresponds to layer I, both tCBV and volume have significant decreases on the control side and whisker trimming significantly reduces both tCBV and volume decreases over 30 days on the trimmed side. **(E)** Total CBV (tCBV) and volume changes over 30-day trimming period in the depth that corresponds to layer IV of the barrel cortex. Both tCBV and volume have no significant changes on the control side and there is no significant whisker trimming effect on tCBV or volume changes over 30 days on the trimmed side. **(F)** Changes in total CBV (tCBV) is tightly correlated with changes in volume over the 30-day trimming period at the depth that corresponds to layer I of the barrel cortex (*R* = 0.7187, *p* = 0.0038). **(G)** Changes in total CBV (tCBV) is independent of changes in volume over 30-day trimming period at the depth that corresponds to layer IV of the barrel cortex (*R* = 0.0045, *p* = 0.9878).

We have developed a series of new tools for processing and analyzing mouse MRI, including cortical surface reconstruction, thickness estimation, layer parcellation and image visualization (See Methods), and have combined them with previously-developed applications. We applied these collective tools to map CBV-fMRI across the mouse cortex and within its cortical layers (Fig 1B). Validating these tools, the CBV pattern visualized across the mouse cortex concords with its known *ex vivo* neuron density architecture [27, 28], and more importantly, the approach is able to map the metabolic organization of the barrel cortex across its layers in living mice (Fig 1A).

We followed an established experimental protocol for induced dendritic spine loss [23]. We implemented the unilateral whisker cutting protocol in a group of young mice, and derived CBV and volumetric measures from the barrel cortex layers before and 4 weeks after whisker cutting. Whole brain analysis revealed that the greatest difference in CBV localized to the vicinity of the barrel cortex (Fig 1C). Using our new analytic tools, we segmented the volumes of the specific barrel cortex layers, allowing us to measure CBV in each layer in two ways. The first is %CBV, which is an estimate of CBV density within a unit volume of brain, similar to CBV density estimates used in prior functional imaging studies [9, 29]. However, as established for other functional readouts of the brain, for example neurons or synapses, density measures introduce potential biases [5, 30–32]. ‘Total’ measurements are superior, typically generated by multiplying density by regional volume [30, 31]. We therefore used this approach to also generate total CBV (tCBV) for each layer.

We focused on layer I and layer IV, layers in which spine loss occurs and does not occur, respectively, with this paradigm [23]. Confirming our prediction, layer I but not layer IV had a loss in both CBV and volume in the contralateral barrel cortex after unilateral whisker cutting (Fig 1D and 1E). As anticipated, tCBV was superior to %CBV in mapping these changes (S1 Table in S1 Appendix). Moreover, these changes were strongly correlated with each other in layer I, as well as layer II, but not in layer IV (Fig 1F, 1G and S1 Table in S1 Appendix).

In secondary analysis, we investigated all layers of the barrel cortex, and found that some layers had decreases of CBV or volume (S1 Table in S1 Appendix). Besides layer I, only layer V and layer II/III, two additional layers that contain synapses, had a decrease in both. Besides layer I, however, a significant but less reliable correlation between both measures was found only in layer II/III.

These studies suggest that when it is known that a condition is not associated with neuronal death, as is the case with normal aging [5], an MRI-based measure sensitive to dendritic spine loss should fulfill two criteria: a decrease in regional tCBV and regional volume, and a correlation between both MRI variables.

### Brain regions vulnerable and resistant to normal aging from 20–72 years of age

From a recently completed study of normal aging in humans, we then investigated which cortical and hippocampal region best fulfilled these criteria in an age-related manner. One hundred and four subjects were recruited to participate in an aging study at Columbia University (the ‘Columbia Cohort’) in whom CBV-fMRI and structural MRI were acquired with a 3T MRI scanner. Subjects ranged from 20–72 years of age and were screened for cognitive defects. Contrast-enhanced CBV and volumetric measurements were derived as previously described (see Methods). Guided by the mouse studies, tCBV was generated as above. Six subjects were excluded from CBV analysis due to missing or poor quality scans. In exploratory analysis, the most reliable age-related changes were observed in the left hemisphere, as described previously [8, 9, 33]. We, however, cannot exclude technical reasons for this lateralizing effect and handedness information was unavailable. All analyses were conducted from measurements derived from the left brain.

#### CBV-fMRI

**Fig 2:**
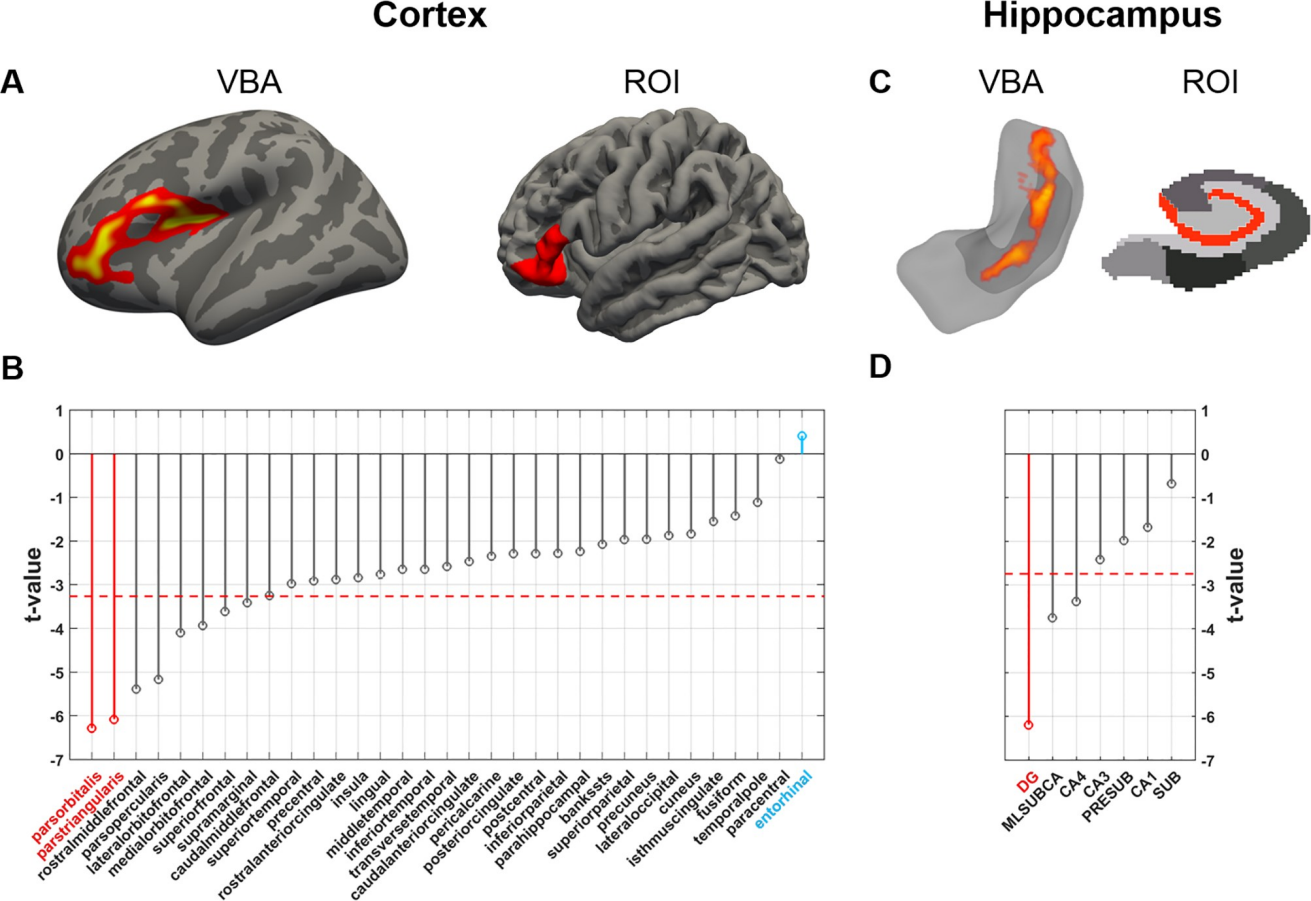
Mapping aging with CBV-fMRI from 20–72 years of age. **(A)** A vertex-based analysis of the cortex (VBA; left image) and a region-of-interest analysis across cortical regions (ROI; right image) identified the greatest age-related decrease in cerebral blood volume (CBV) in the inferior frontal gyrus. **(B)** The t-value distribution of age-related CBV decreases across cortical regions shows that two regions of the inferior frontal gyrus (indicated in red, the pars orbitalis and the pars triangularis) are most reliably vulnerable to aging. The entorhinal cortex (indicated in blue) was found most resistance to aging. The dashed red line indicates the t-value threshold at *α* = 0.05 adjusted for Šidák multiple comparison. **(C)** A voxel-based analysis of the hippocampus (VBA; left image) and a region-of-interest analysis across hippocampal regions (ROI; right image) identified the greatest age-related CBV decrease in the dentate gyrus. **(D)** The t-value distribution of age-related CBV decrease across hippocampal regions, shows that the dentate gyrus (indicated in red) is most reliably vulnerable to aging. The dashed red line indicates the t-value threshold at *α* = 0.05 adjusted for Šidák multiple comparison.

To map age-related CBV changes across the cortex, a vertex-wise linear regression model was used, with tCBV values as the dependent variable, age as the regressor, and gender and intracranial volume (ICV) as covariates. The most reliable age-related tCBV decrease across cortex was observed in the vicinity of the inferior frontal gyrus (Fig 2A). For the complimentary ROI analysis, we used FreeSurfer to parcellate the cortex [34, 35] and applied the linear regression model for each individual region. All FreeSurfer outputs were inspected to ensure that there were no gross topological defects. The only two regions that showed significant age-related CBV decreases at significance level α *=* 1e-6, after applying a Šidák correction, were two constituent subregions of the inferior frontal gyrus (pars orbitalis: *t* = -6.28, *p* = 1.04e-8; pars triangularis: *t* = -6.08, *p* = 2.61e-8, two-tailed, *N* = 98) (Fig 2B; we use t-values that emerged from the linear regression model to illustrate the ordering across regions, but the ordering does not differ using other metrics such as effect sizes). Unexpectedly, the entorhinal cortex was the cortical region that was least affected by aging (*t* = 0.411, *p* = 0.682, two-tailed, *N* = 98) (Fig 2B).

To map age-related CBV changes within the hippocampus, a voxel-based analysis was performed using the same linear regression model. The most reliable age-related difference was observed in the dentate gyrus (Fig 2C). For the complementary ROI analysis, we used the latest FreeSurfer hippocampal subregion segmentation technique [36], and by applying the same linear regression model to each hippocampal subregion, the dentate gyrus (granule cells/molecular layer/dentate gyrus, ‘GCMLDG’) was the dominant site of age-related CBV decrease (*t* = -6.19, *p =* 1.53e-8, two-tailed, *N* = 98) (Fig 2D).

#### Volumetric MRI

**Fig 3:**
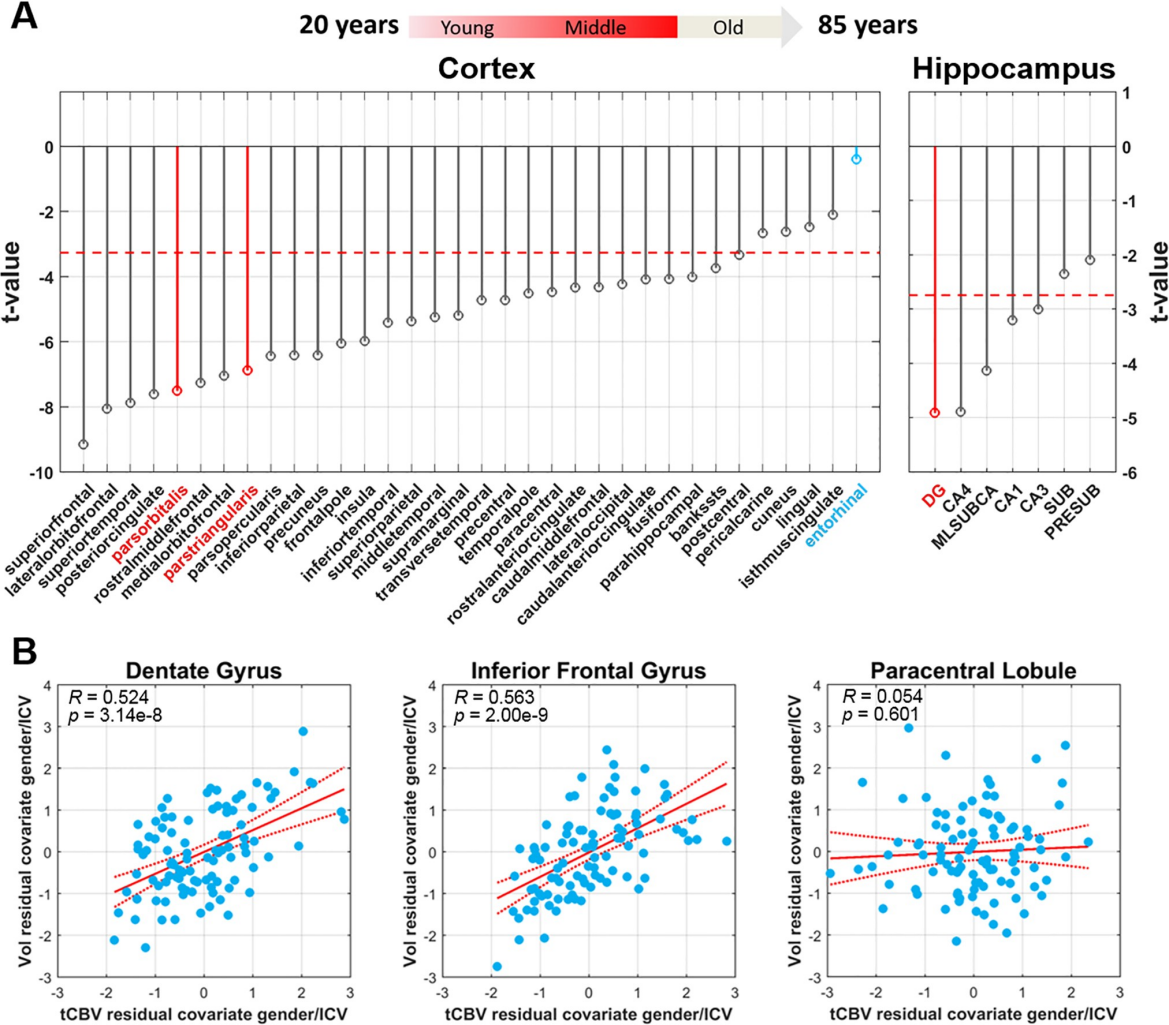
Mapping aging with volumetric MRI from 20–72 years of age and its relationship to CBV-fMRI. **(A)** The t-value distribution of age-related decrease in volume, measured by structural MRI, across cortical regions in 20-72-year old healthy subjects (indicated in red is the arrow illustration above) While not most reliably affected, volume in regions of the inferior frontal gyrus (indicated in red) show a decrease with age. The volume of the entorhinal cortex (indicated in blue) was found most resistance to aging. The dashed red line indicates the t-value threshold at *α* = 0.05 adjusted for Šidák multiple comparison. **(B)** A significant relationship between CBV and volume observed for the dentate gyrus and the inferior frontal gyrus, a joint MRI biomarker which best correlates with dendritic spine loss, but not for the entorhinal cortex.

To map age-related volumetric changes across the cortex and within the hippocampus, the linear regression model was applied to the volumes of each individual cortical region and hippocampal subregion derived by structural MRI. Across the cortex, subregions of the inferior frontal gyrus were associated with age (pars orbitalis: *t* = -7.50, *p* = 2.62e-11; pars triangularis: t = -6.88, p = 5.38e-10, two-tailed, N = 104), although, unlike for CBV, their volumes were not the ones most reliably associated with age (Fig 3A). As with CBV, the entorhinal cortex was the cortical region least correlated to age (*t* = -0.445, *p* = 0.657, two-tailed, N = 104). Within the hippocampus, the dentate gyrus was again the region differentially associated with age (*t* = -5.04, *p* = 2.08e-6, two-tailed, N = 104).

#### CBV-volume relationships

Guided by the mouse studies, we found a correlation between tCBV and volume in the dentate gyrus (*R* = 0.524, *p* = 3.14e-8, two-tailed, N = 98) and the inferior frontal gyrus (*R* = 0.563, *p* = 2.00e-9, two-tailed, N = 98) (Fig 3B).As such, the combined MRI profile is reflective of dendritic spine loss. In an exploratory analysis, as in mice, some regions fulfilled one criterion but not both (S5 Fig in S1 Appendix). For example, paracentral lobule showed a relationship of age with volume (*t* = -4.48, *p* = 2.13e-5, two-tailed, N = 98) but not with tCBV (*t* = -0.123, *p* = 0.902, two-tailed, N = 98) or a reliable correlation between tCBV and volume (*R* = 0.054, *p* = 0.601, two-tailed, N = 98) (Fig 3B).

### Brain regions vulnerable and resistant to normal aging from 62–85 years of age

We next set out to broaden the age span to include subjects in older age groups. However, because of the high prevalence of preclinical AD in this age-range we needed to rely on CSF biomarkers, particularly the most reliable tau/Aβ_1–42_ ratio, to attempt and correct for this potential confound. From the ‘Alzheimer’s Disease Neuroimaging Initiative’ (ADNI), we were able to identify 80 subjects who were cognitively normal at baseline and for 4 years of clinical follow-up, and who had baseline CSF data, among which we include 52 subjects who were CSF biomarker negative for AD.

**Fig 4:**
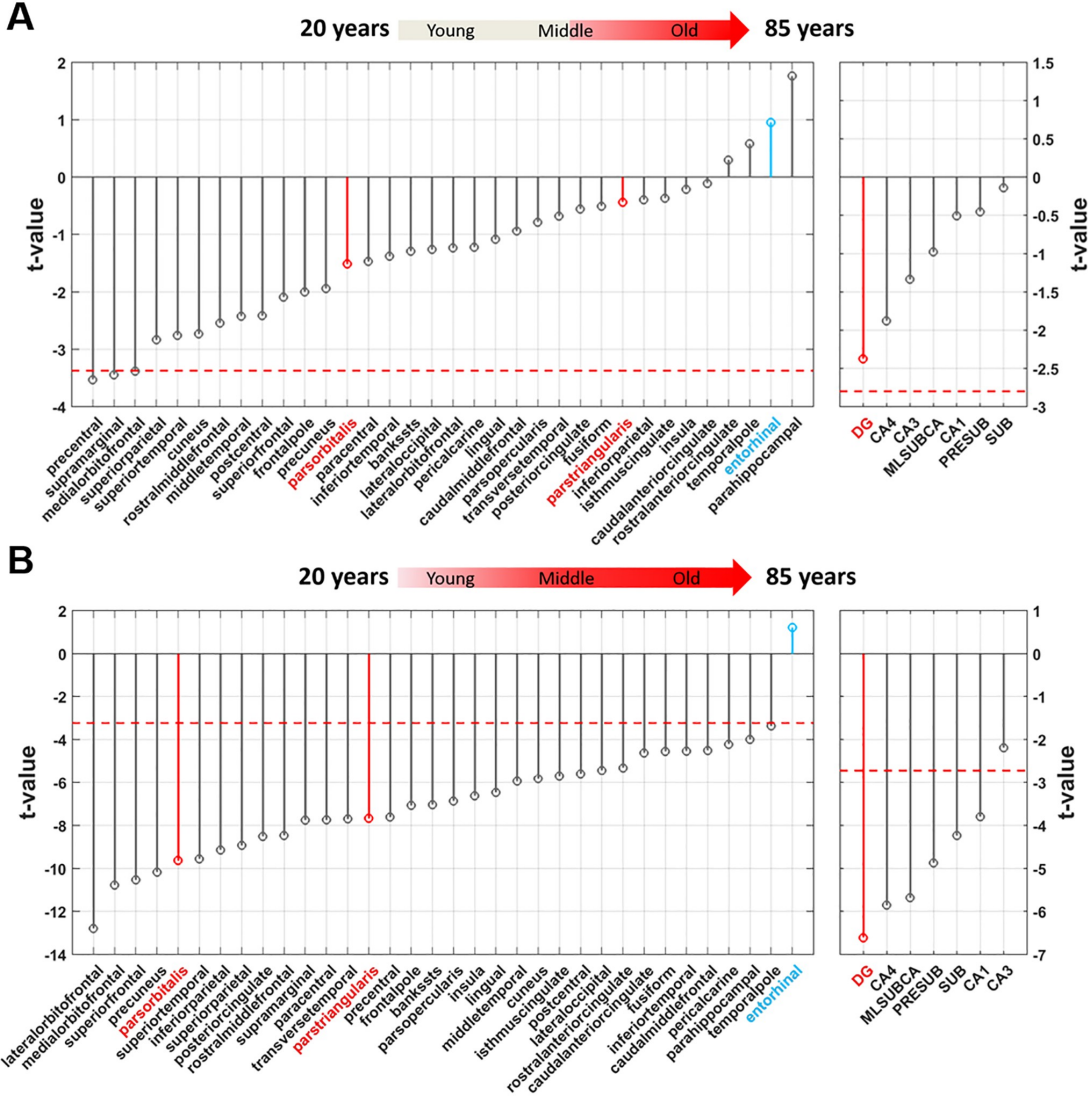
Mapping aging with volumetric MRI from 62–85 years of age and across the age-span. **(A)** The t-value distribution of age-related decreases in volume across cortical and hippocampal region in Alzheimer’s-free 62–85 year old subjects (indicated in red in the arrow illustration above), shows that the dentate gyrus is most vulnerable to aging (although not crossing threshold of multiple comparisons) and the inferior frontal gyrus (indicated in red, left graph) is not reliably associated with aging. The entorhinal cortex (indicated in blue, left graph) is the region least affected by aging. The dashed red line indicates the t-value threshold at *α* = 0.05 adjusted for Šidák multiple comparison. **(B)** The t-value distribution of age-related decrease in volume across cortical and hippocampal region in Alzheimer’s-free subjects across the full age-span, of both the ADNI and Columbia aging cohort, (indicated in red in the arrow illustration above), shows that the dentate gyrus (indicated in red, right graph) is most vulnerable to aging and the inferior frontal gyrus (indicated in red, left graph) is reliably associated with aging. The entorhinal cortex (indicated in blue, left graph) is the region least affected by aging. The dashed red line indicates the t-value threshold at *α* = 0.05 adjusted for Šidák multiple comparison.

We generated cortical and hippocampal volumes as with the Columbia Cohort and then applied the same linear regression model to each volume (Fig 4A). Across the cortex, regions of the inferior frontal gyrus were unrelated to age in this age-span, but here again the entorhinal cortex was one of the regions least related to age. Within the hippocampus, the dentate gyrus was again the region most reliably associated with age (*t* = -2.37, *p* = 0.0217, two-tailed, N = 52), however, likely because of the constrained age-range, the effect in the dentate gyrus did not meet the Šidák-corrected threshold for significance at α = 0.05.

### Brain regions vulnerable and resistant to normal aging across the age-span

We then combined the volumetric data of the Columbia and ADNI cohorts in order to map aging effect independent of AD across the full adult lifespan—from young adulthood, through midlife, to old age. By mapping the age-related volumetric differences (Fig 4B) the most notable result is that among all brain regions investigated, the entorhinal cortex is the region least associated with normal aging (*t* = 1.21, *p* = 0.227, two-tailed, N = 156). Not surprisingly, based on the previous analyses, the dentate gyrus was the hippocampal region most reliably associated with age (*t* = -6.61, *p* = 6.24e-10, two-tailed, N = 156). The inferior frontal gyrus, while not the region most related, was still associated with age (pars orbitalis: *t* = -9.63, *p* = 1.99e-17; pars triangularis: *t* = -7.67, *p* = 1.96e-12, two-tailed, N = 156).

**Fig 5:**
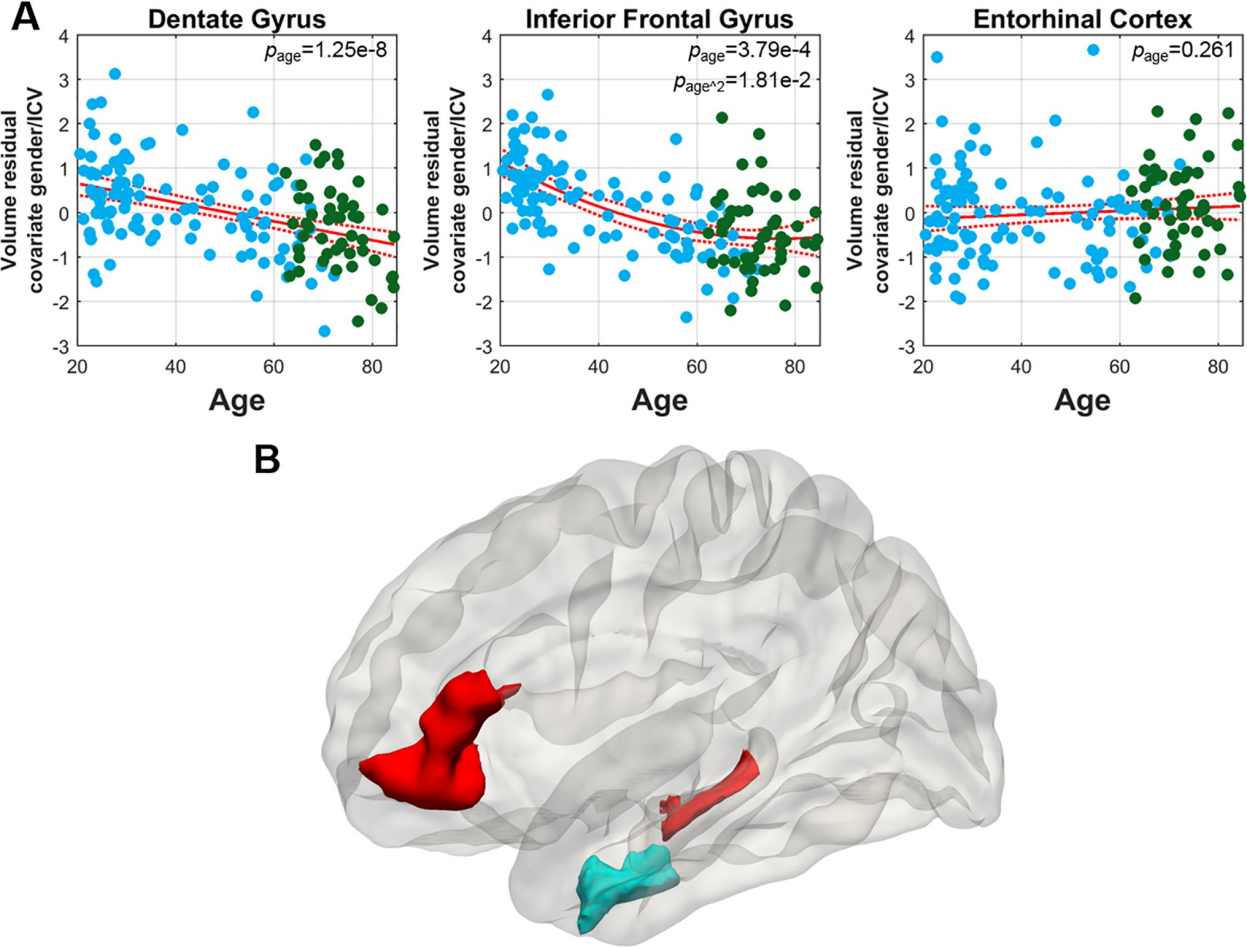
Trajectories of brain regions vulnerable and resistant to normal aging across the age-span. **(A)** The aging trajectory of dentate gyrus volume (left image) shows a linear decrease across the age-span. The trajectory of inferior frontal gyrus volume (middle image) shows a curvilinear decrease. The trajectory of entorhinal cortex volume (right image) shows that it unaffected by aging across the age-span. **(B)** A graphic summary of the two regions differentially vulnerable to normal aging, the dentate gyrus and the inferior frontal gyrus (red), and the region most resistant to normal aging, the entorhinal cortex (blue).

Next, we relied on the combined dataset to explore the trajectories across the life span of the dentate gyrus, the inferior frontal gyrus, and the entorhinal cortex. While the association between age and dentate gyrus was linear across the full age span (*p*_age_ = 1.25e-8), the inferior frontal gyrus was best modeled by a quadratic curve (*p*_age_ = 3.79e-4, *p*_age^2_ = 1.81e-2) (Fig 5A), where the effect of aging on this brain region appears to taper off in the older years. The entorhinal cortex was best modelled as a flat line across the full age span (*p*_age_ = 0.261) (Fig 5A).

### An exploratory longitudinal analysis

ADNI affords the opportunity to investigate structural MRI changes over a few-year period. Although normal aging occurs slowly over the course of many years, we nevertheless considered it interesting to explore this restricted longitudinal dataset.

From the 52 subjects who were CSF biomarker negative for AD we further identified 47 subjects who have at least two follow-up MRI scans among months 6, 12, 24, and 48 visits and pass quality check. We performed linear mixed effects model with gender, ICV, baseline age, visits (time from baseline) as fixed effects, individual intercept and slope as random effects. In general, this restricted longitudinal analysis agreed with the more definitive cross-sectional analyses, showing the dentate gyrus is the second most significant hippocampal region with longitudinal age-related volume decrease, regions of the inferior frontal gyrus show moderate longitudinal age-related volume decrease, and entorhinal cortex is one of the regions that show least significant atrophy (S7 Fig in S1 Appendix).

## Discussion

In anticipation of our human aging studies, we first performed an MRI study in mice using a paradigm that is known to induce dendritic spine loss in specific cortical layers. From these mouse studies we learned that while both CBV-fMRI and structural MRI are sensitive to spine loss, a concordant change in both enhances this sensitivity. Additionally, we learned that for CBV-fMRI mapping, a derived tCBV is a better parameter than derived %CBV. We acknowledge that these MRI measure might also be observed in the setting of neuronal death. However, in conditions like healthy aging, in which it is known *a priori* that there is minimal neuronal death, the concordant fMRI-MRI measure is superior to each individually in acting in enhancing detection of dendritic spine loss.

Informed by these mouse studies, we then used MRI to derive tCBV and volume/thickness measures in a normal aging study. In support of the conclusion that this aging cohort was not dominantly confounded by occult disease, no age-related changes was observed in the entorhinal cortex, a region known to be affected first in AD [29, 37–39] and has CBV [29] and volume [40] loss in even preclinical AD. In contrast, the dentate gyrus within the hippocampus, and the inferior frontal cortex across the cortex, were the two regions that showed the most reliable age-related decrease in the mouse-derived MRI correlate of dendritic spine loss.

While we consider this primary human study the most rigorous in achieving our goal of mapping brain aging without AD, we nevertheless considered it important to try and map aging without AD across a broader age-span. This goal, however, presents a number of challenges. When investigating aging in older age groups, it becomes necessary to rely on AD biomarkers to try and exclude preclinical AD, and for this reason we set out to accomplish this goal by turning to ADNI. The advantages of ADNI is that it acquires CSF AD biomarkers as well as structural MRI in a large number of healthy elders. Unfortunately, ADNI does not acquire high-resolution functional imaging variants akin to CBV-fMRI, and so we cannot apply the highest MRI-based criteria for spine loss, one based on both a high-resolution functional measure and volume. Nevertheless, because previous studies suggest that structural MRI can be sensitive to dendritic spine loss, a conclusion supported by our mouse studies, we considered an analysis of structural MRI alone potentially informative. Another challenge is how to use CSF AD biomarkers to attempt and reduce the confound of preclinical AD, and we opted to use tau/Aβ_1–42_ because it is now generally considered the most reliable CSF biomarker of AD [26]. An additional advantage of ADNI is that it acquires structural MRI longitudinally over a few-year period. Although aging occurs slowly and over decades, ADNI affords the opportunity to determine whether longitudinal changes—albeit in a smaller number of subjects and over a very narrow time span—generally agrees with the larger and longer cross-sectional analysis.

Despite all the challenges, and despite the fact that structural MRI is not the most reliable marker of spine loss, the secondary ADNI analyses and the combined ADNI-Columbia analysis largely supported some of the findings observed in the more definitive primary human study, as did the exploratory longitudinal analysis. Collectively, two findings emerged as most consistent across all analyses. First, within the hippocampus the dentate gyrus is the region most dominantly affected by normal aging, a finding that agrees with previous imaging studies in non-human mammals [14, 15], and also with a recent postmortem analysis of the disease-free human hippocampus [41].

Second, across the cortex and hippocampus, the entorhinal cortex was the brain region that was most resistant to normal aging. We consider this a more novel and unexpected finding, as the entorhinal cortex is a region that is differentially vulnerable to AD [29, 37–39]. The imperviousness of the entorhinal cortex to normal aging was previously suggested by studies that have imaged aging animal models [14, 15], but these focused exclusively on the hippocampal formation—a term that encompasses both the entorhinal cortex and the regions of the hippocampus. We note that while another cross-sectional structural MRI study showed that, across the full sweep of the life span, the entorhinal cortex was one of the least affected by aging, a more restricted longitudinal analysis in the same study suggested that the entorhinal cortex was a region that showed a lot of thinning with age [43]. In contrast to our longitudinal structural MRI study, however, the previous study used Aβ_42_ alone as an exclusionary biomarker, and relied on a smaller sample who were followed for only 1–2 years. These factors can account for this one discrepancy.Similarly, other studies have shown a marked loss of thickness after the age of 60[42] as well as preservation of entorhinal cortex and other nearby structures [44] but without CSF exclusionary criteria, Recent studies have showed that using both CSF Aβ_42_ and tau biomarkers is more precise in excluding AD [45], and that CSF tau in particular is associated with entorhinal cortex atrophy in incipient disease [46]. Informed by this insight, our longitudinal analysis not only included more subjects scanned for a longer period of time, but more importantly our analysis used both CSF Aβ_42_ and tau in addressing the preclinical AD confound. Nevertheless, because these longitudinal analysis track, in the best scenario, individuals for only a few years, we consider them only exploratory.

The third observation that emerged from our collective studies is that, across the cortex, the inferior frontal gyrus is also region targeted by normal aging. Here, however, we found the greatest discrepancies between the first Columbia cohort, and the secondary ADNI studies, which by necessity was only performed on structural data. In the Columbia cohort, findings suggested that the inferior frontal cortex was the region that was most reliably affected by normal aging. This agrees with previous functional imaging studies [8, 9, 47, 48], but since we used a high-resolution fMRI technique we were able to map, for the first time, age-related dysfunction in the dentate gyrus and the inferior frontal gyrus in the same cohort, using a validated biomarker of spine loss. In contrast to the Columbia cohort, findings from the ADNI cohort showed that while the inferior frontal gyrus is affected by aging, it was not the region most reliably affected. One explanation for this discrepancy emerged from the combined Columbia-ADNI analysis, which suggested that in contrast to the linear age-related decrease in the dentate gyrus, age-related changed in inferior frontal gyrus tapers off in older age. Another explantion for the disprepency, was the in contrast to the study in the Columbia cohort, in which we had access to both CBV and structural data, in the ADNI studies only structural data was available. Structural data might be sensitive to other regional contituents, such as glia, which might confound the results. For either reason, our results reduce our confidence that the inferior frontal gyrus is differentially linked to aging, and other cortical regions should also be considered.

Our results have a number of implications. First, since the inferior frontal gyrus and the dentate gyrus are regions involved in cognitive function, and the latter a site of adult neurogenesis [49], our study confirms that normal aging targets our cognitive faculties more than sensory or motor abilities. Second, our results elucidate the cognitive phenotype of normal aging versus AD. Previous studies documented that cognitive performance on tasks that have been linked to the inferior frontal gyrus [50, 51] and the dentate gyrus typifies aging [17, 52]. At the same time, cognitive performance on tasks that are linked to the entorhinal cortex are among those most sensitive to AD [29, 53] but resistant to aging [17].

Identifying vulnerable and resistant regions, and mapping the aging trajectories of the vulnerable regions, can be used for understanding cellular and molecular mechanisms that underlie normal aging, however these must be taken in light of differences in neuronal laminar distributions and morphology between species [54]. In previous studies we relied on the spatial pattern of age-related dysfunction in the hippocampal formation, and the temporal pattern of age-related dentate gyrus dysfunction to isolate the histone-binding protein, RbAp48, as a molecular correlate of age-related hippocampal dysfunction in human postmortem tissue [16]. This finding was validated in mouse models, showing that RbAp48 plays causal role in age-related memory decline [16]. Genetic polymorphisms in the gene encoding for RbAp48 have been linked to hippocampal-dependent age-related memory [16], as have other genetic polymorphisms, for example, polymorphisms in *BDNF* [55]. Future studies can now rely on the spatio-temporal pattern we identified in the inferior frontal gyrus and other frontal cortex regions to potentially isolate molecular causes of age-related frontal cortex dysfunction.

The most interesting implication of our study, we believe, relates to the entorhinal cortex. We note that the entorhinal cortex can be further parsed along its longitudinal and transverse axes, and future studies might be able to pinpoint its site of greatest resistance. Is it a coincidence that of all regions, the one that is apparently most resistant to aging is also the region most vulnerable to AD? It seems unlikely. We propose that the mechanisms that account for entorhinal cortex resistance is inversely linked to its vulnerability to disease. We predict that any molecular, cellular or network feature that can explain why the entorhinal cortex is differentially resistant to aging would provide insight into why this region is vulnerable to AD.

## Methods

### Experimental animals

All animal procedures and experiments were performed in accordance with national guidelines (National Institutes of Health) and approved by the Institutional Animal Care and Use Committee of Columbia University. 7 male young (<3 months) C57bl/6 wild type mice were selected for our study. Mice were group-housed of 3 or 4 with 12 h on/off light cycles. Whisker trimming was performed daily in the late afternoon for 30 days by cutting the mystacial vibrissae of the right whisker-pad to skin level with a pair of scissors under anesthesia. Mice were anesthetized using a gaseous mixture of 30% O_2_, 70% N_2_ and 2–3% isoflurane at 1 liter/min air flow, via a nose cone. After whisker trimming, mice were immediately returned to their original cage to allow quick recovery and free movement.

### Human subjects

Participants in this study are from two cohorts: Columbia life-span cohort and ADNI normal aging cohort. The top chart in S1 Fig in S1 Appendix summarizes the demographic information of the two cohorts with different MRI modalities. Further detail on subject inclusion and exclusion criteria can be found in S2 Fig in S1 Appendix.

#### Columbia life-span cohort

For Columbia life-span cohort, subjects are healthy adults between age 20 and 72, recruited from the Columbia University Medical Center campus. MRI acquisition was part of a Columbia University Medical Center Institutional Review Board approved study, with explicit written consent obtained from each participant. All subjects were screened through neuropsychological testing and found to be free of dementia at the time of testing.

#### ADNI normal aging cohort

The details about the ADNI study design can be found in the ADNI website (http://adni.loni.usc.edu/study-design). We identified normal aging subjects by excluding potential preclinical AD subjects using longitudinal follow-up and CSF criteria. To satisfy longitudinal follow-up criterion, subjects were evaluated as cognitively normal at baseline and stayed cognitively normal during the follow-up. The follow-up period has to be at least 4 years. Following the official documentation from ADNI [56], we identified N = 52 normal aging subjects with the concentration ratio of total tau and Aβ_1–42_ (t-tau/Aβ_1–42_) lower than the cut-off value 0.39. For the longitudinal analysis, among the 52 subjects, we further constrain the analysis to N = 47 subjects with at least two follow-up MRI scans and passing quality check.

### Structural MRI processing

For the human study, the T1-weighted structural images were processed using FreeSurfer 6.0, generating cortical parcellation [34, 35] and hippocampal subregions segmentation [36] in the individual structural image space. The primary hippocampal subregions labeled include presubiculum (PRESUB), subiculum (SUB), CA1, CA3, CA4 (hilus), granule cell molecular layer of DG (DG), molecular layer of subiculum and CA fields (MLSUBCA). The list of cortical regions can be found in the parcellation protocol documented in [35].

For the mouse study, the T2-weighted structural images were analyzed using an in-house developed MATLAB software suite, MouseStream [57]. MouseStream provided a processing and analysis pipeline consisted of a pre-processing phase followed by a tri-step cortical parcellation approach.

At the pre-processing phase, all three T2-weighted images were isotropically upsampled with cubic B-spline interpolation, intensity-normalized, skull-stripped, linearly co-registered [58]. A gadolinium enhanced MRI (GEMRI) image for each animal was calculated as the grayscale-inverted median of all three processed T2-weighted images.

Briefly, the tri-step cortical parcellation approach consists of template creation, cortex delineation and curved cortical coordinate system construction. More details can be found in S1 Appendix, Methods.

To create the template, a population average of GEMRI images was constructed through an iterative process, by averaging the co-registered GEMRI images over multiple cycles using a symmetric diffeomorphic algorithm with cubic-spline interpolation [59].

Cortex delineation was achieved using inter-modality atlas-to-atlas registration that registered an *ex vivo* atlas to the *in vivo* GEMRI template to obtain labels for the *in vivo* images. Specifically, an *ex vivo* MRI T2*-weighted mouse brain atlas [60] was registered to the GEMRI template by maximizing their mutual information using mincANTS (http://www.picsl.upenn.edu/ANTS/). Pia surface and white matter surface of the GEMRI template were defined from the co-registered *ex vivo* label map.

In the last step of cortical parcellation, similar to an *ex vivo* study described in the Allen Institute technical white paper: Allen Mouse Common Coordinate Framework [27], a curved cortical coordinate system was developed to enable the integration, comparison and visualization of mouse brain information from different cortical depths *in vivo*. Streamlines connecting pia and white matter surfaces can be used to facilitate the annotation of the entire cortex or a specific cortical region. In addition, cortical thickness can also be calculated. To obtain the cortical thickness map, cortical label map of the template image was firstly warped into the subject space to determine the cortex borders for each animal, followed by generating cortical streamlines. The cortical thickness at each point on the pia surface was calculated as the integrated length of the corresponding streamline.

### CBV-fMRI processing

CBV-fMRI processing followed the steps applied in previous studies [17, 29, 61]. More details can be found in S1 Appendix, Methods. In multi-variate analyses, for the human study, individual structural images were registered into template space using a symmetric diffeomorphic algorithm [59]. Individual CBV images were linearly registered into the individual structural image space. The CBV images were registered to the template space with the linear transformation matrix and the diffeomorphic transformation field. For the mouse study, the CBV images were registered to the template space by forward applying the corresponding linear transformation matrix and the diffeomorphic transformation field derived from the GEMRI registration. In ROI analyses, for both human subjects and mice, total CBV (tCBV) measures the total amount of cerebral blood volume in an anatomically defined ROI and was calculated as the product of the regional %CBV density and the ROI volume. In voxel/vertex-based analyses, tCBV measures the voxel/vertex-wise local total amount of cerebral blood volume and is the voxel/vertex-wise product of CBV and volumetric measures.

### Data analysis of mice

#### Percentage CBV density voxel-based analysis in the cortex

Voxel-wise paired t-test was performed between CBV images of time 1 (baseline) and CBV images of time 2 (30-day after whisker trimming) using SPM8 (http://www.fil.ion.ucl.ac.uk/spm/software/spm8), to locate significant %CBV decreases. The results were thresholded at p<0.05 and spatial cluster size larger than 20.

#### Total CBV ROI analysis in the barrel cortex

Based on the previous studies [23] and our %CBV VBA results, the tCBV ROI analysis was only focused on the barrel cortex at different cortical depths. Using the technique described above, the structural template of GEMRI images was processed through the MouseStream pipeline to automatically segment cortical regions of the whole brain and delineate the barrel cortex into eight evenly divided depths. In the co-registered template space, ROI tCBV values were calculated as the product of the regional averaged %CBV and the volume at each depth of the barrel cortex for both hemispheres. For each depth, a paired t-test was used for the data analysis with ‘time’ (time 1 versus time 2) as the repeated-measures factor, and ‘hemisphere’ (trimmed-side versus control-side) as the related-groups factor for all animal subjects. We used one-tailed paired t-test to compare the longitudinal changes of ROI tCBV of the trimmed-side versus the control-side and test the hypothesis that tCBV decrease on the trimmed-side was less (*p*<0.05).

#### Volume ROI analysis in the barrel cortex

Same as the tCBV ROI analysis, volumes of each depth in the barrel cortex were also analyzed for all animal subjects.

#### Correlation analysis between ROI total CBV and volume in the barrel cortex

For the correlational analyses, we compared the Pearson’s correlation between longitudinal changes of tCBV and volume at each depth of the barrel cortex of both hemispheres.

### Data analysis of human subjects

#### Total CBV voxel-based analysis in the hippocampus

For each voxel, the tCBV value is the product of %CBV and the Jacobian determinant, which measures the relative volume change warping from unit volume element into the template space. Voxel-wise linear regression with age as regressor, gender and ICV as covariates was performed using SPM8. The results were thresholded at *p*<0.05 and spatial cluster size larger than 10.

#### Total CBV vertex-based analysis in the cortex

We used vertex-based analysis for cortical tCBV analysis. The cortical surface in individual structural image space was reconstructed using FreeSurfer [62]. The co-registered CBV image was projected onto the reconstructed cortical surface along the surface normal with projection fraction from 0 to 1 in the step of 0.1. The projected maps were then averaged into a single map representing the surface-based cortical CBV. The individual surfaces along with the projected CBV maps were registered into the FreeSurfer *fsaverage* space for inter-subject analysis. Cortical volume maps in the same space were generated using FreeSurfer, where the volume measure at each vertex is the volume of the local truncated tetrahedron. The tCBV at each vertex is the product between the %CBV and volume. Vertex-wise linear regression with age as regressor, gender and ICV as covariates, was performed using custom scripts. The results were thresholded at p<0.01 and spatial cluster size larger than 500.

#### Total CBV ROI analysis

The cortical parcellation and hippocampal subregion segmentation were registered into the CBV-fMRI image space with the transformation matrix from registering individual CBV image into the individual structural image space. Regional %CBV is the average %CBV within each ROI. Regional tCBV is the product of the regional %CBV and the ROI volume. Linear regression with age as regressor, gender and ICV as covariates was performed for each ROI. t-values that directly emerged from the linear regression model were used to illustrate the ordering of the aging effect across regions. It should be noted that the frontal pole labeled in the segmentation protocol was not fully covered in a small portion of the CBV-fMRI scans because of the FOV thus were not included in the results.

#### Volume ROI analysis

In the cross-sectional volume ROI analysis, for each ROI, we used linear regression with ROI volume as dependent variable, age as regressor, gender and ICV as covariates. In the regression analysis of the aging trajectory of IFG, since we observed a curvilinear relationship, we fit a linear regression with both age and age squared as regressors, gender and ICV as covariates. We further compared the Akaike information criterion (AIC) between the regression model with and without the second order term to select the best-fitted model. t-values were used to illustrate the ordering of the aging effect across regions.

In the longitudinal volume ROI analysis, we performed linear mixed effects model with gender, ICV, baseline age, visits (time from baseline) as fixed effects; individual intercepts and slopes as random effects. We reported the t-values of the effect of longitudinal visits on regional volumes from the model.

### Correlation analysis between ROI total CBV and volume

We performed Pearson partial correlation analysis between ROI total CBV and volume with gender and ICV as covariates, and reported the correlation coefficient in S5 Fig.

## Supporting information

S1 Data (XLSX)

S1 Appendix Supplementary information text. (DOCX)

S1 File (XLSX)
