# Supplementary material for: Brain regions vulnerable and resistant to aging without Alzheimer’s disease
Source: PLoS One. 2020 Jul 29;15(7):e0234255. doi: 10.1371/journal.pone.0234255 (PMC7390259; doi:10.1371/journal.pone.0234255)
Supplement: S1 Appendix — (DOCX) [file pone.0234255.s002.docx]

Supplementary Information Text

Supplementary Method

## MRI Acquisition. For the CBV-fMRI human study, we used a steady-state contrast enhanced CBV technique as previously described (1, 2). MRI scans were acquired with a Philips Achieva 3.0 T MRI scanner using an 8-channel SENSE head coil. In each scan session, a T1-weighted structural scan (TR = 6.7 ms, TE = 3.1 ms, FOV = 240 × 240 × 192 mm^3^, voxel size = 0.9 × 0.9 × 0.9 mm^3^) was first acquired using a Turbo Field Echo (TFE) gradient echo (GRE) sequence; a pair of un-scaled T1-weighted images (TR = 6.7 ms, TE = 3.1 ms, FOV = 240 × 240 × 162 mm^3^, voxel size = 0.68 × 0.68 × 3 mm^3^) were acquired afterwards with a bolus injection of gadolinium contrast agent in between. For the ADNI dataset, the details of the MRI acquisition protocols (3) can be found in the project website^[[1]](#footnote-1)^. For the mouse study, two sets of images were acquired using CBV-fMRI protocols as previously described (1, 4) for each animal at baseline and 30 days after whisker trimming. A Bruker BioSpec 94/20 (field strength, 9.4 T; bore size, 20 cm) horizontal small animal MRI scanner with software ParaVision 6.0.1 (Bruker BioSpin, Billerica, MA, USA) and a 23-mm 1H circularly polarized transmit/receive capable mouse head volume coil were used for the imaging. Mice were anesthetized using the medical air and isoflurane (3% volume for induction, 1.1-1.5% for maintenance at 1 liter/min air flow, via a nose cone). A flowing water heating pad was used to maintain the body temperature at around 37°C. Sterile eye lubricant was applied after each scan. Three T2-weighted structural images were acquired before, within and 53.5 min after intraperitoneal injections of the contrast agent Gadodiamide (Omniscan; GE Healthcare, Princeton, NJ, USA) at the dosage of 10 mmol/kg for CBV mapping, with the first T2-weighted scan as the pre-contrast image and the last T2-weighted scan as the post-contrast image. T2-weighted images were acquired with a fast-spin echo acquisition (TR = 2,300 ms, effective TE = 45 ms, RARE factor = 16, FOV = 16.2 × 21.0 × 8.6 mm^3^, voxel size = 60 × 60 × 200 µm^3^, scan time = 35 m 34 s).

## MouseStream. This section details the tri-step cortical parcellation approach consisted of template creation, cortex delineation and curved cortical coordinate system construction. To create the template, each of the 14 GEMRI images (i.e., 7 mice by 2 time points) was first flipped across the mid-sagittal plane. The registration template was created using ANTs^^[[2]](#footnote-2)^^. A population average of 14 x 2 = 28 GEMRI images (with the flipped images used as additional inputs) through an iterative process, by averaging the co-registered GEMRI images over multiple cycles using a symmetric diffeomorphic algorithm with cubic-spline interpolation (7). The symmetric diffeomorphic registration provided an invertible transformation field composed of a 12-parameter affine transformation matrix and a diffeomorphic transformation map which allows each GEMRI image to be warped into the template space and vice versa. To achieve cortex delineation, after registration, label map from the ex vivo atlas was warped into the in vivo template space with nearest neighbor interpolation for cortical delineation of in vivo images. In addition, pia and white matter surfaces of the in vivo template image were created from the label map as the outer surface of the cerebral hemispheres and the white-gray matter junction. For cortical parcellation, in the template space, after the borders of cortex were defined by cortex delineation as described previously, Laplace’s equation was solved between pia and white matter surfaces resulting in intermediate equi-potential surfaces (8). Streamlines (with 500 points/streamline) were computed starting from points belonging to the pia surface by finding the steepest descent path through the equi-potential field. Information at different cortical depths can then be projected along the streamlines onto the pia surface to allow integration, comparison or visualization. Both in the template space and the subject space, the pia surface can be further deformed for the purpose of a better visualization of the whole cortex into the 2D Euclidean space through surface flattening while conserving relative cortical surface areas (64).

## Structural MRI processing. For human longitudinal analysis, The MRI scans were processed with FreeSurfer longitudinal pipeline (5, 6).

## CBV-fMRI processing. For the human study, processing steps include registration of the pre-contrast and the post-contrast T1-weighted scans, subtraction of the co-registered post-contrast and pre-contrast scans, and CBV value normalization with the mean signal in the superior sagittal sinus. For the mouse study, processing steps include registration of pre-contrast and the post-contrast T2-weighted scans, log-transformation of the ratio between the co-registered pre-contrast and post-contrast scans, and CBV value normalization with the mean signal in the posterior cerebral artery, as delineated by a blinded rater. The raw CBV values are %CBV measures in the unit volume defined by the voxel.

## Percentage CBV density VBA and ROI analysis. The VBA and ROI analyses using %CBV are similar to the VBA and ROI analyses of tCBV. In each linear regression analysis, we used %CBV as dependent variable, age as regressor, and gender as covariate.

Thickness ROI analysis in the cortex. We also explored using cortical thickness measures instead of cortical volumes. For each ROI, we used linear regression with mean thickness of the ROI as dependent variable, age as regressor, and gender as covariate. t-values that directly emerged from the linear regression model was used to illustrate the ordering of the aging effect across regions.


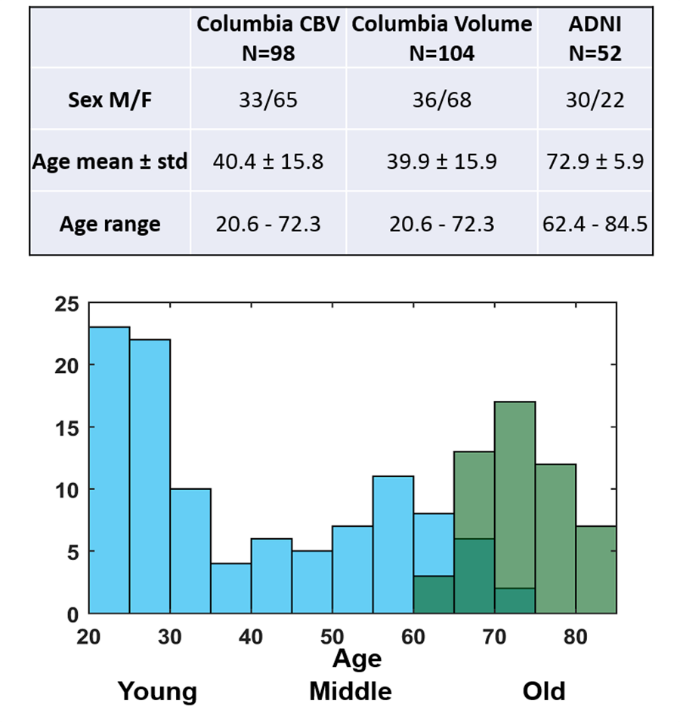


**Fig. S1.** Demographics and age distribution of participants in the aging studies. The chart shows the demographic information of the Columbia and ADNI cohorts. The bar graph below shows the age distribution of the Columbia (blue), the ADNI cohort (green), and the overlap (dark green), together covering the adult age-span.


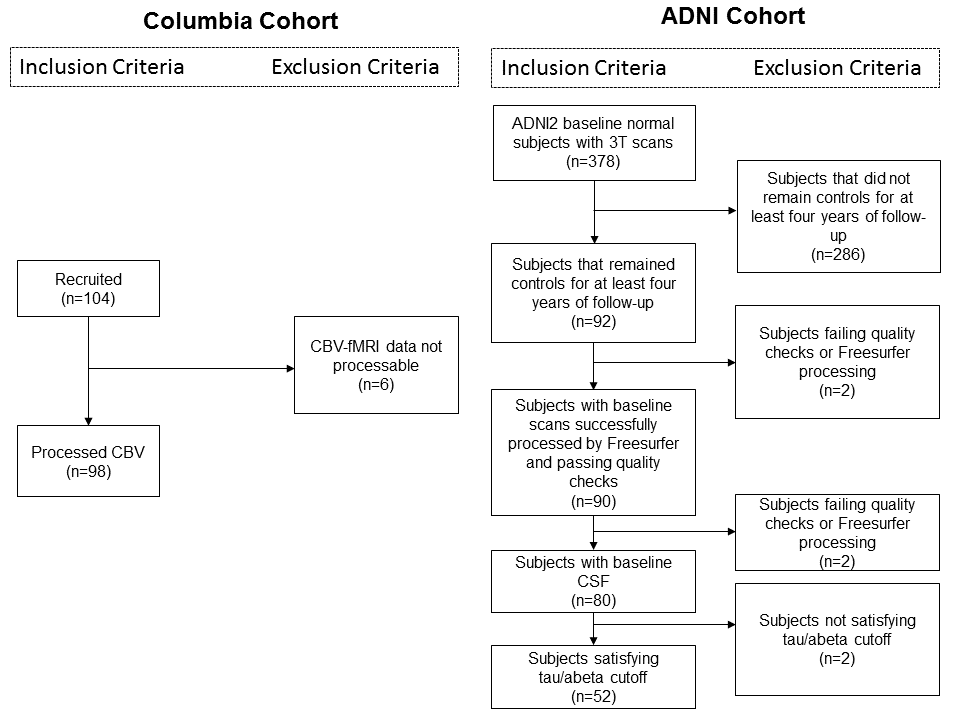


Fig. S2. Recruitment charts for both the Columbia and ADNI cohorts.


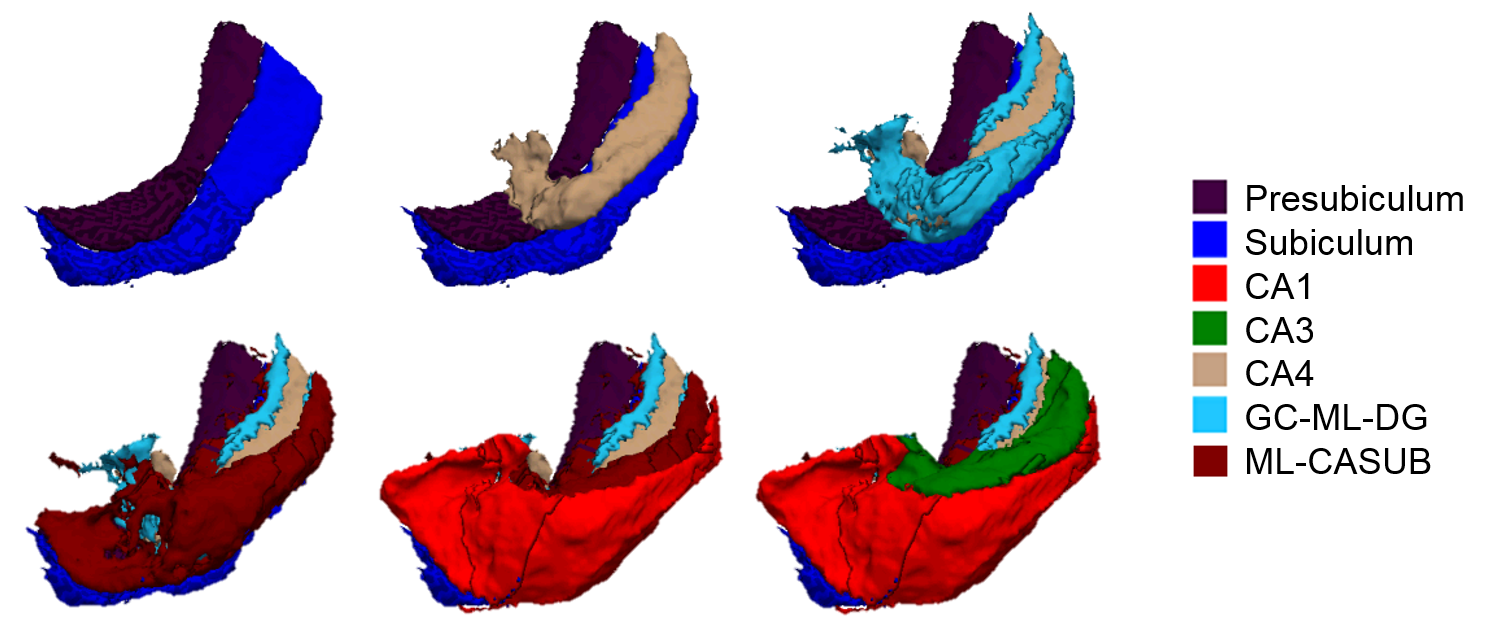


Fig. S3. The hippocampal subregions of the template image segmented using FreeSurfer.


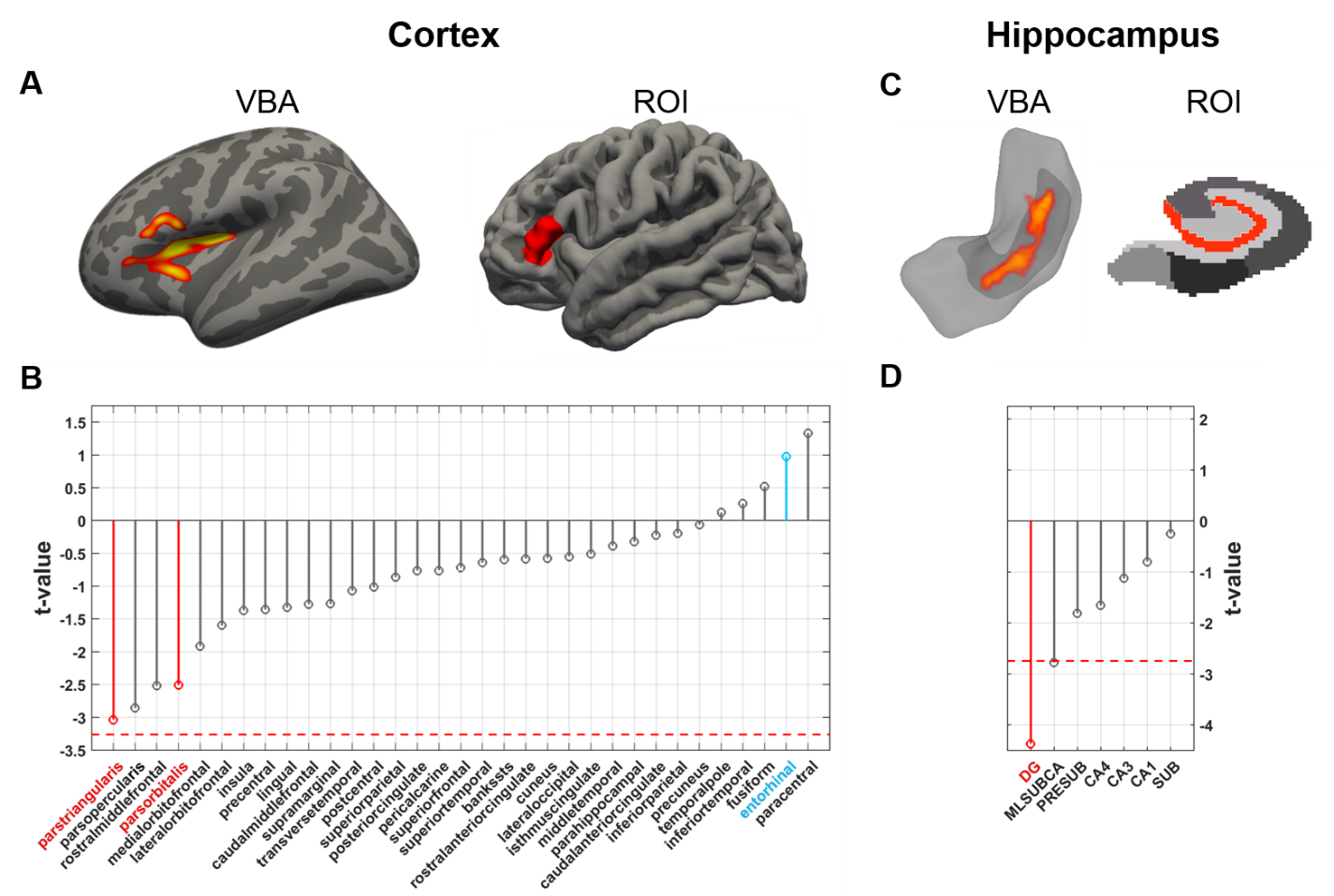


Fig. S4. Mapping aging with CBV-fMRI from 20-72 years of age. The corresponding figure of Figure 3 using %CBV.

(A) A vertex-based analysis of the cortex (VBA; left image) and a region-of-interest analysis across cortical regions (ROI; right image) identified the greatest age-related decrease in %CBV in the inferior frontal gyrus.

(B) The t-value distribution of age-related %CBV decrease across cortical regions shows that two regions of the inferior frontal gyrus (indicated in red, the pars orbitalis and the pars triangularis) are still among the most vulnerable regions to aging. The entorhinal cortex (indicated in blue) was found to be one of the most resistant regions to aging. The dashed red line indicates the t-value threshold at α=0.05 adjusted for Šidák multiple comparison.

(C) A voxel-based analysis of the hippocampus (VBA; left image) and a region-of-interest analysis across hippocampal regions (ROI; right image) identified the greatest age-related %CBV decrease in the dentate gyrus.

(D) The t-value distribution of age-related %CBV decrease across hippocampal regions, shows that the dentate gyrus (indicated in red) is most reliably vulnerable to aging. The dashed red line indicates the t-value threshold at α=0.05 adjusted for Šidák multiple comparison.


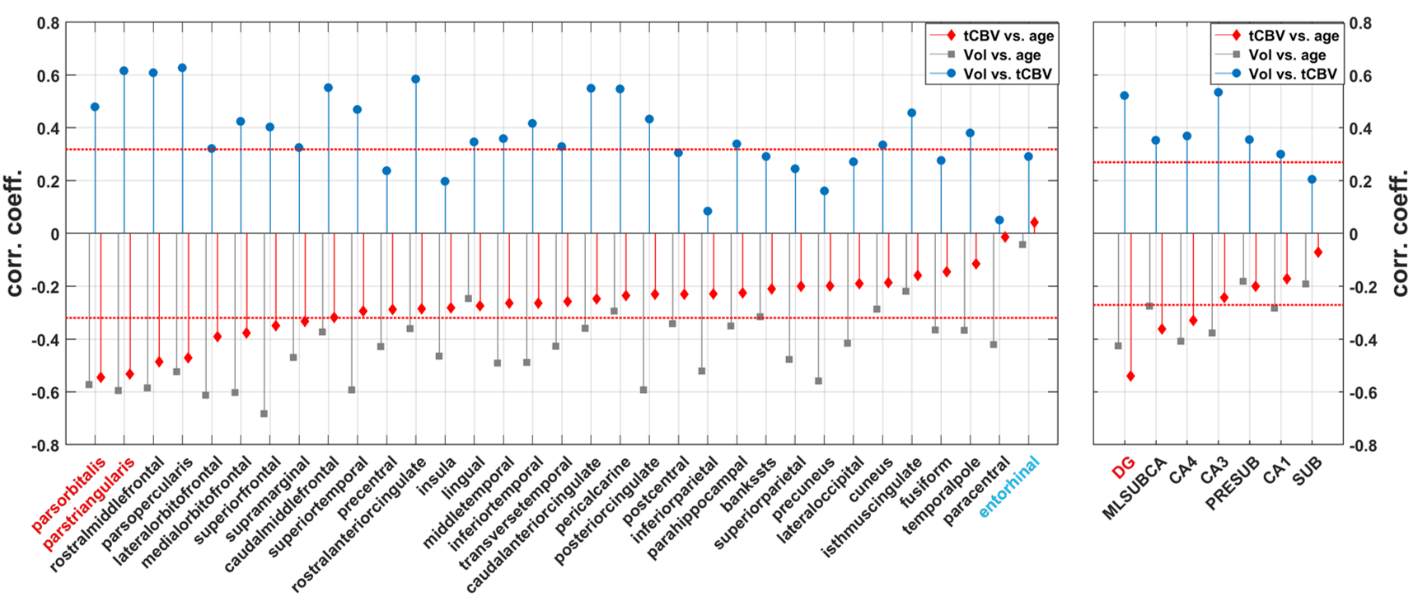


Fig. S5. A summary graph of the partial correlation coefficients of tCBV versus age, volume versus age, and tCBV versus volume across the full age-span, with gender and ICV as covariates. The dashed red line indicates the correlation coefficient threshold at α=0.05 adjusted for Šidák multiple comparison.


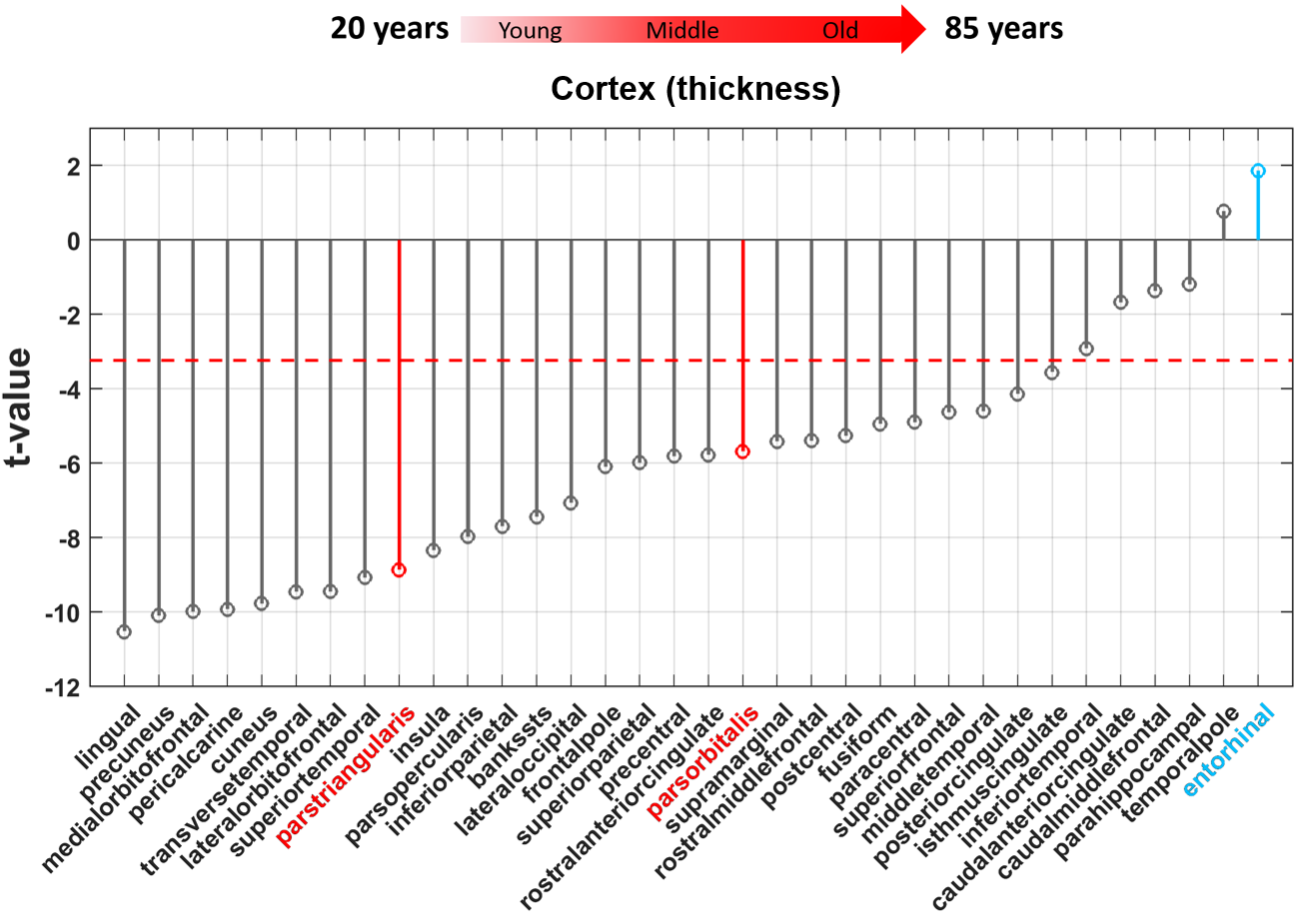


Fig. S6. The t-value distribution of age-related decrease in cortical thickness in Alzheimer’s-free subjects across the full age-span, shows that the inferior frontal gyrus (indicated in red) is reliably associated with aging. The entorhinal cortex (indicated in blue) is the region least affected by aging. The dashed red line indicates the t-value threshold at α=0.05 adjusted for Šidák multiple comparison.


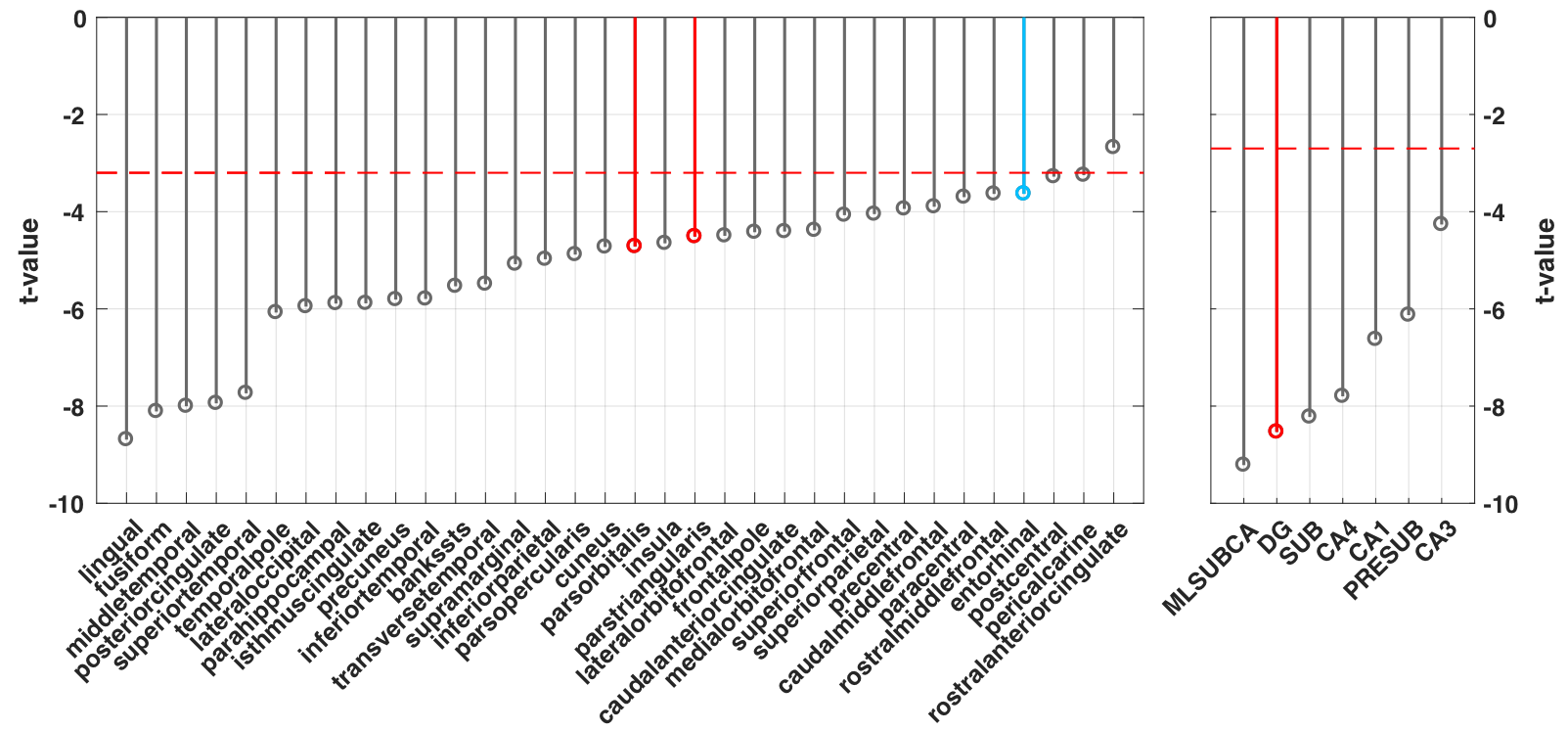


Fig. S7. Mapping longitudinal age-related change with volumetric MRI from 62-85 years of age. The t-value distribution of longitudinal age-related volume decline across cortical and hippocampal regions from 62-85 years of age with baseline tau/Aβ1-42 under cut-off, shows that dentate gyrus is the second most significant hippocampal subregion, while entorhinal cortex is one of the regions that show least significant atrophy. The dashed red line indicates the t-value threshold corresponding to α = 0.05 adjusted for Šidák multiple comparison.

Table S1. The volume changes (ΔVol), total CBV changes (ΔtCBV), percentage CBV changes (Δ%CBV) on the trimmed side (T) and control side (C) over 30-day trimming period, and the p-values for the one-tailed t-test between trimmed side and control side. The last two columns show the correlation coefficient between volume change (ΔVol) and tCBV change (ΔtCBV), and the p-value of the correlation. ** indicates p<0.005, * indicates p<0.05.


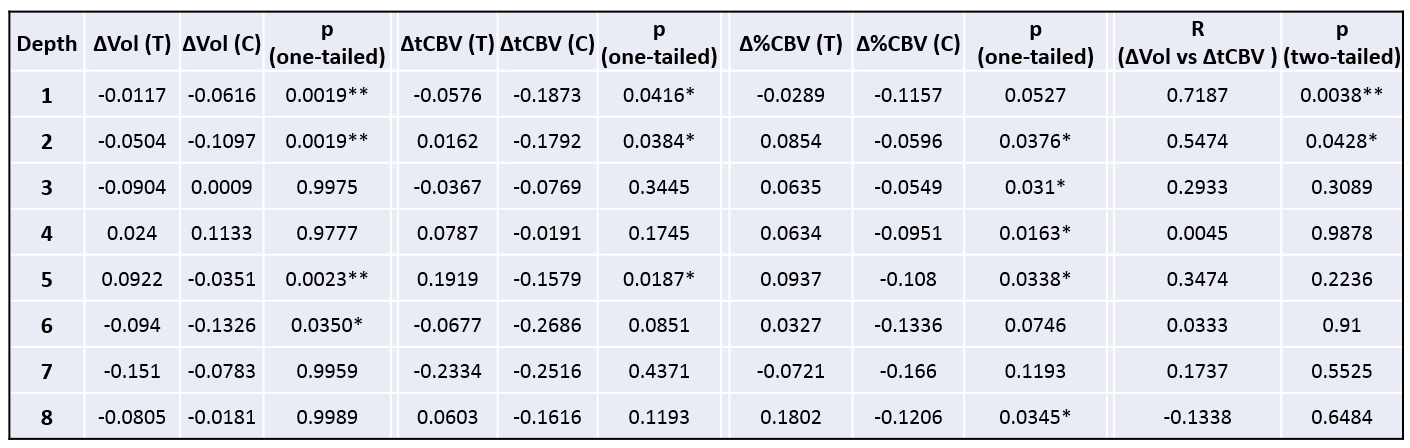


**References**

1. U. A. Khan *et al.*, Molecular drivers and cortical spread of lateral entorhinal cortex dysfunction in preclinical Alzheimer's disease. *Nature Neuroscience* **17**, 304-311 (2014).

2. A. M. Brickman *et al.*, Enhancing dentate gyrus function with dietary flavanols improves cognition in older adults. *Nature Neuroscience* **17**, 1798-1803 (2014).

3. C. R. Jack *et al.*, The Alzheimer's Disease Neuroimaging Initiative (ADNI): MRI Methods. *Journal of Magnetic Resonance Imaging* **27**, 685-691 (2008).

4. H. Moreno, F. Hua, T. Brown, S. Small, Longitudinal mapping of mouse cerebral blood volume with MRI. *NMR in Biomedicine* **19**, 535-543 (2006).

5. J. E. Iglesias *et al.*, Bayesian longitudinal segmentation of hippocampal substructures in brain MRI using subject-specific atlases. *NeuroImage* **141**, 542-555 (2016).

6. M. Reuter, N. J. Schmansky, H. D. Rosas, B. Fischl, Within-subject template estimation for unbiased longitudinal image analysis. *NeuroImage* **61**, 1402-1418 (2012).

7. B. B. Avants *et al.*, A reproducible evaluation of ANTs similarity metric performance in brain image registration. *NeuroImage* **54**, 2033-2044 (2011).

8. S. E. Jones, B. R. Buchbinder, I. Aharon, Three-dimensional mapping of cortical thickness using Laplace's equation. *Human Brain Mapping* **11**, 12-32 (2000).

1. http://adni.loni.usc.edu/adni-mri-methods/ [↑](#footnote-ref-1)
2. http://www.picsl.upenn.edu/ANTS/ [↑](#footnote-ref-2)
